# Ruthenium(II) Complex with 3,4-Methylenedioxy Cinnamic Acid Induces Cell Cycle Arrest at G0/G1 and Apoptosis via ROS Generation and Bioenergetics Disruption in Non-Small Cell Lung Cancer Cells

**DOI:** 10.1021/acsomega.5c00526

**Published:** 2025-07-01

**Authors:** Guilherme Álvaro Ferreira-Silva, Caio Cesar Candido, Graciana Yokota Garavelli, Carolina Girotto Pressete, Ester Siqueira Caixeta, Angelica Ellen Graminha, Marília Imaculada Frazão Barbosa, Antônio Carlos Doriguetto, Marisa Ionta, Alexandre Ferro Aissa

**Affiliations:** † Institute of Biomedical Science, 74347 Federal University of Alfenas, 37130-000 Alfenas, Minas Gerais, Brazil; ‡ Institute of Chemistry, Federal University of Alfenas, 37130-001 Alfenas, Minas Gerais, Brazil; § Institute of Chemistry, São Paulo State University, 14800-060 Araraquara, São Paulo, Brazil

## Abstract

Lung cancer is the leading cause of cancer-related deaths globally, with nonsmall cell lung cancer (NSCLC) being the most prevalent type. Cisplatin and its derivatives are commonly used in classical chemotherapy, but their therapeutic efficacy is limited, and they came with significant side effects. The targeted therapies and immunotherapy have improved survival rates for certain groups of patients. However, overall survival for NSCLC remains very low, which contributes to the high mortality rate. Thus, the search for effective and affordable drugs is ongoing. In this context, ruthenium-based complexes have been extensively studied as promising anticancer agents. In our previous studies, three ruthenium­(II) complexes containing cinnamic acid derivatives with ligands were synthesized and characterized. We demonstrated that the complex [Ru­(*trans*-4-(trifluoromethyl)­cinnam)­(dppb)­(bipy)]­PF_6_ effectively inhibited the proliferation of melanoma cells with either an activating *N-Ras* mutation or a *TP53* inactivation mutation. Therefore, in this study, we aimed to evaluate the antitumor potential of complex [Ru­(3,4-cinnam)­(dppb)­(bipy)]­PF_6_, named CINNAM, against nonsmall cell lung cancer cells A549 (*TP53* wild type; *K-Ras* mutated) and H1299 (*TP53* mutated; *N-Ras* mutated). CINNAM selectively targeted cancer cells over normal cells, with A549 cells showing higher sensitivity to the CINNAM compared to H1299 cells. CINNAM effectively reduced the proliferation of A549 cells by inducing cell cycle arrest at the G0/G1 phase. CINNAM modulated the expression profiles of key cell cycle regulators, *CDKN1A*, *CCND1*, and *CCNE2*. Further, we demonstrated that the cytotoxic activity of CINNAM is linked to its pro-oxidant and pro-apoptotic properties. The interactions of the complex with *ct*-DNA (calf thymus DNA) were analyzed, and based on the *K*_b_ value, the complex present intermediate affinity for DNA. In conclusion, CINNAM exhibits potent antiproliferative and pro-apoptotic effects on NSCLC cells, surpassing those observed with cisplatin.

## Graphical abstract

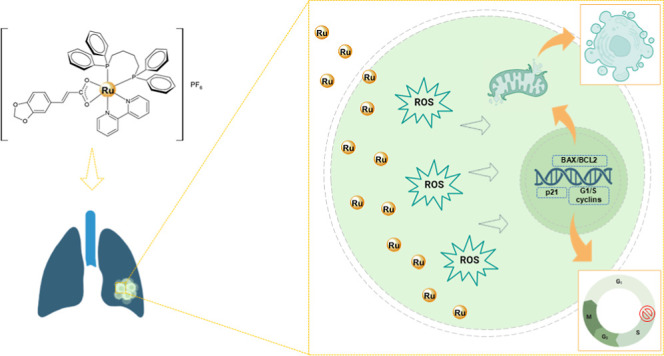

## 1 Introduction

The pursuit of effective therapies for lung cancer has spanned decades.^1^ Significant strides have been made, particularly following the identification of mutations that can be therapeutically targeted, laying the foundation for targeted therapies. These advances are largely responsible for the reduction in lung cancer mortality observed between 2013 and 2016, highlighting the impact of ongoing research and improved treatments.^2^ Unfortunately, despite these advancements, lung cancer continues to be the leading cause of cancer-related deaths worldwide.^3^ It is important to highlight that while targeted therapies are typically accessible in developed countries, they remain largely unavailable in developing nations.^1^ In addition, many patients are inherently resistant to, or eventually develop resistance against drugs used in classical chemotherapy and targeted therapy.

Thus, the ongoing pursuit of antitumor drugs focuses on discovering new prototypes with diverse mechanisms of action that can be produced with low financial investments. The goal is not only to overcome tumor resistance processes but also to ensure that these new therapies can benefit patients across all socioeconomic backgrounds, particularly in regions where targeted therapies are limited or unavailable.

Remarkably, since the 1970s, cisplatin, a metal coordination compound known as *cis*-diamminedichloroplatinum­(II), along with its derivatives, has remained the cornerstone of treatment for lung cancer.^4–6^ Its therapeutic effectiveness stems from its unique mechanism of action, which involves forming cross-links in the DNA of tumor cells, thereby inhibiting DNA replication and transcription and inducing apoptosis.^7^

The limitations of cisplatin have prompted exploration into other metal-based compounds as potential antitumor agents, including ruthenium complexes.^8^ Although numerous ruthenium complexes have been developed, none have yet achieved clinical success as effective antitumor agents. Some, such as NAMI-A,^9^ KP1019,^10^ NKP1339,^11^ and TLD1443,^12^ have been tested in clinical trials with modest outcomes, while others have shown antitumor potential in vitro and in vivo.^13^ These findings have spurred the continued synthesis and testing of new complexes against tumor cell lines.

Ruthenium complexes are often synthesized with molecules that have complementary chemical and biological properties. These additions, such as cinnamic acid, enhance the original compound’s solubility, stability, and biological selectivity. This natural compound, extracted from cinnamon bark, consists of a benzene ring, an alkene double bond, and an acrylic acid functional group. This structure allows for modifications that yield bioactive agents with improved therapeutic efficacy. Derivatives of cinnamic acid have shown significant potential as antitumor agents.^14^

In our previous study, we synthesized a series of ruthenium­(II) complexes containing cinnamic acid derivatives (3,4-methylenedioxy cinnamic acid; *trans*-4-(trifluoromethyl)­cinnamic acid, and *trans*-4-chloro-cinnamic acid). We previously demonstrated that the complex with *trans*-4-(trifluoromethyl)­cinnamic acid inhibited cell cycle progression at the G1 phase in melanoma cells, induced apoptosis, and disrupted the actin cytoskeleton network, contributing to decrease cell viability, proliferation, and migration.^15^ Therefore, herein we explored the antitumor properties of ruthenium­(II) complex containing 3,4- methylenedioxy cinnamic acid obtained in our previous study, on nonsmall cell lung cancers (A549, and H1299) once some genetic profiles of melanoma cells are common with cells derived from NSCLC.

## 2 Material and Methods

### 2.1 Cell Lines and Culture Conditions

The NSCLC A549 and H1299 cells, and the normal lung fibroblasts (IMR-90) were purchased from the Rio de Janeiro Cell Bank and cultured in Dulbecco’s modified Eagle’s medium (DMEM)/F12, (catalog no. D8900, Sigma-Aldrich, Saint Louis, MO, USA) supplemented with 10% fetal bovine serum (FBS, Cultilab, SP, Brazil). The cultures were maintained in an incubator at 37 °C with a controlled atmosphere (95% air and 5% CO_2_), and subculturing was performed regularly every 2 or 3 days.

### 2.2 Synthesis of Ruthenium­(II) with 3,4-Methylenedioxi Cinnamic Acid

The synthesis of the ruthenium­(II) compound used in the present study was described in detail by Negreti et al.,^15^ and Graminha et al.^16^ Briefly, the reaction of 3,4-methylenedioxy cinnamic acid with the precursor *cis*-[RuCl_2_(dppb)­(bipy)] (where bipy = 2,2′-bipyridine and dppb = 1,4-bis­(diphenylphosphino)­butane) was employed to obtain the respective complex [Ru­(3,4-cinnam)­(dppb)­(bipy)]­PF_6_ (Figure 1). The compound was characterized by elemental analysis, ^31^P­{^1^H}, ^13^C­{^1^H} and ^1^H NMR spectroscopy, UV–vis and IR spectra, molar conductance, and cyclic voltammetry. Furthermore, the synthesized complex was analyzed by liquid chromatography, confirming the purity of 97.9% (see Supporting Information, Figure S1), in agreement with elemental analysis [Anal. (Calc) [RuC_48_H_43_N_2_O_4_P_2_]. PF_6_: exp. (calc) 56.16 (56.53); H, 4.24 (4.25); N, 2.84 (2.75)%.

**Figure 1:**
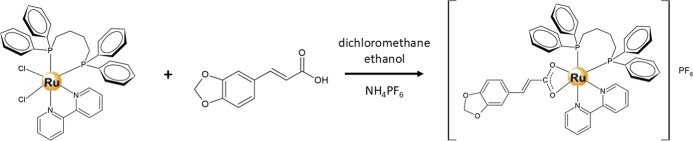
[Ru­(3,4-cinnam)­(dppb)­(bipy)]­PF_6_ synthetic route.

CINNAM compound was characterized, and results are in agreement with reported by Negreti et al.^15^ and Graminha et al.^16^ ^31^P­{^1^H} NMR spectra in CH_2_Cl_2_ revealed doublets for CINNAM, which is due to the magnetically different phosphorus atoms as expected (Figure 1). The ^31^P­{^1^H} NMR chemical shifts were different from those of the *cis*-[RuCl_2_(dppb)­(bipy)] starting material, suggesting that the presence of 3,4-methylenedioxy cinnamic acid coordinated to the metal shifted the electron density of the phosphorus atoms of the dppb ligand. Additionally, in the ^1^H NMR spectrum of the 3,4-methylenedioxy cinnamic acid ligand, a signal corresponding to a singlet of the O–H group proton was observed in 10.5 ppm. These signals are not observed in the spectra of the complex, indicating the deprotonation of the hydroxyl group after its coordination to the metal (see Supporting Information, S2–S4).

The infrared spectrum of the complex confirms the presence of 3,4-methylenedioxy cinnamic acid ligand coordinated to the metal. Additionally, the electronic spectra show three bands in the UV region, assigned to intraligand transitions by means of comparison with the free ligand (dppb and 3,4-methylenedioxy cinnamic acid). One band observed in the visible region results from a metal-to-ligand charge transfer transition, probably involving both diimine and 3,4-methylenedioxy cinnamic acid (see Supporting Information, S5–S6).

In the cyclic voltammogram, a quasi-reversible process due to the redox pair Ru­(II)/Ru­(III) was observed. The difference observed between them and the precursor may be due to the different stereochemistry found for them. The *E*_1/2_ value found for the complex was considerably more anodic than that observed for the precursor [RuCl_2_(bipy)­(dppb)], indicating that the ruthenium center is more stable after coordination of 3,4-methylenedioxy cinnamic acid compared with the precursor (see Supporting Information, S7).

Time-dependent ^31^P­{^1^H} NMR experiments were performed to evaluate the stability of the complexes in DMSO + DMEM solution, over the period from 0 to 48 h. The ^31^P­{^1^H} NMR analysis reveal that the complex was stable during this time length (Figure 2).

**Figure 2:**
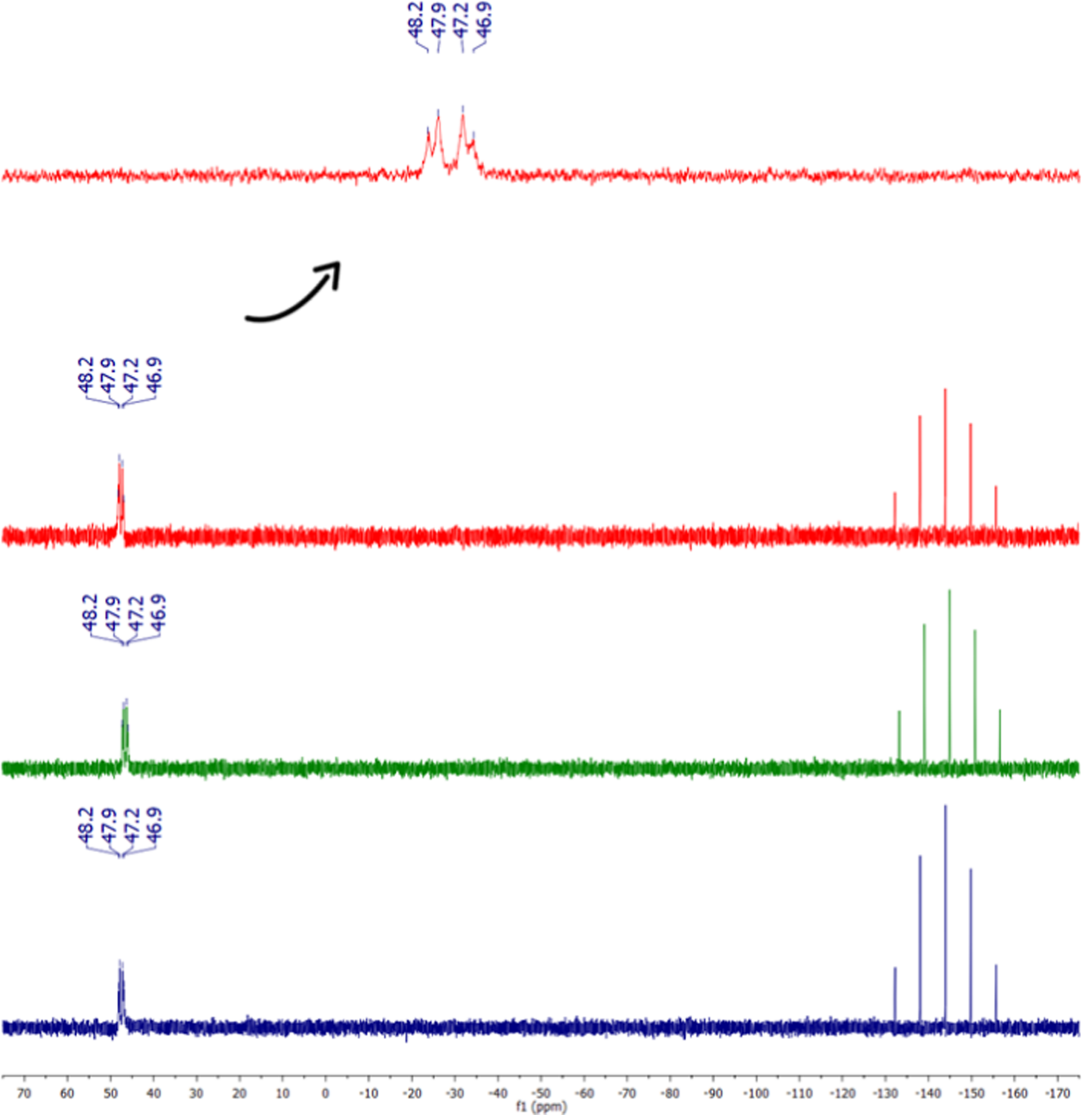
Compound stability by ^31^P­{^1^H} NMR spectroscopy: 0 h (red), 24 h (green) and 48 h (blue), DMSO + DMEM, with D_2_O capillary.

### 2.3 Cell Viability Evaluation

#### 2.3.1 Colorimetric Assay Based on the Conversion of Tetrazolium Salt to Formazan

Cells were seeded in 96-well plates at a density of 1 × 10^4^ cells/well. The substances were tested at concentrations of 1, 10, 25, 50, and 100 μM for 24 h. We chose these concentrations based on our previous study with the same ruthenium complex.^15^ After the treatment, 10 μL of MTS reagent (catalog no. #G3580, Promega, Madison, WI, USA) was added to each well. After 4 h of incubation at 37 °C, the samples were analyzed using a spectrophotometer at 490 nm. IC_50_ values (the concentration required to inhibit 50% of growth) were determined using Calcusyn software (Biosoft, Ferguson, MO, USA).

#### 2.3.2 Trypan Blue Exclusion Test

Cells were seeded in 35 mm plates at a density of 2 × 10^5^ cells/plate. After 24 h (adhesion), the cells were treated with CINNAM at concentrations of 2 and 4 μM for 24, 48, and 72 h. On the day of analysis, viable and nonviable cells were quantified using a hemocytometer in the presence of 0.4% Trypan Blue dye.

### 2.4 Cytoskeleton Elements Analysis

Cells were seeded on coverslips into 35 mm plates (2 × 10^5^ cells/plate). After treatment, cells were fixed with 3.7% formaldehyde (Sigma) for 30 min. For α-tubulin immunolabeling, cells were permeabilized with Triton X-100 (0.5%) for 15 min. After blocking with 1% BSA, primary antibody (catalog no. T6199, Sigma-Aldrich, 1:100) was incubated overnight. On next day, secondary antimouse IgG-FITC antibody (catalog no. F0257, Sigma-Aldrich, 1:100) was added to the sample and incubated for 2 h. Following, phalloidin–rhodamin conjugated (catolog no. P1951, Sigma-Aldrich, 1:100) incubation (1 h) was performed for actin labeling. Nuclei were stained with DAPI (catalog no. #D3571, Thermo Fisher Scientific, Waltham, MA, EUA) and coverslips were mounted on microscope slides using Vecta-Shield (Vector Laboratories, Newark, CA, USA). All washes were done with PBS. Analyses were performed using a fluorescence microscope (Nikon).

### 2.5 Proliferation Analysis

#### 2.5.1 Clonogenic Assay

Cells were seeded at a low density (500 cells/plate) in 35 mm plates. After adhesion, the cultures were treated with CINNAM at 2 or 4 μM for 24 h. After treatment, the medium was replaced with fresh drug-free medium, and the plates were incubated at 37 °C with 5% CO_2_ for 12 days. Then, the cultures were washed with PBS, and the cells were fixed with methanol PA (Sigma-Aldrich) for 30 min. After drying, the plates were stained with crystal violet for 5 min. Colonies were quantified using a stereomicroscope (20× magnification), and colonies with, at least 50 cells, were counted.

#### 2.5.2 BrdU Incorporation Assay

Cells were seeded in 35 mm plates on coverslips at 2 × 10^5^ cells/plate. After adhesion, the cultures were treated with CINNAM at concentrations of 2 and 4 μM for 24 h. After the treatment, the culture medium was replaced with fresh medium supplemented with BrdU (EMD Biosciences, San Diego, CA, USA) at 100 μM for 1 h. The cells were then fixed with 3.7% formaldehyde for 30 min and treated with 0.5% Triton X-100 for 10 min. The samples were subsequently treated with 1.5 M HCl for 30 min, and after successive washings, they were incubated with anti-BrdU antibody (catalog no. #5292, Cell Signaling, Danvers, MA, 1:100) at 4 °C for 12 h. Antimouse secondary antibody conjugated to FITC (catalog no. F0257, Sigma-Aldrich, 1:100) was then added to the samples for 2 h at room temperature, followed by counterstaining with DAPI (#D3571, Thermo Fisher Scientific, Waltham, MA, EUA) for 30 min at room temperature. The slides were mounted with VectaShield (VectorLabs, Newark, CA, USA) and analyzed under a fluorescence microscope (Zeiss Axio Scope, A1, Oberkochen, Baden-Württemberg, Germany).

#### 2.5.3 Mitotic Index Determination

Cells were seeded in 35 mm plates on coverslips at 2 × 10^5^ cells/plate. After adhesion, the cultures were treated with CINNAM at concentrations of 2 and 4 μM for 24 h. Mitotic cells were counted from fluorescent cytological preparations containing DAPI-stained nuclei. 1000 cells were counted per sample.

### 2.6 Cell Cycle Analysis

Cells were seeded in 35 mm plates at a density of 2 × 10^5^ cells/well. After adhesion, the cells were treated with CINNAM at concentrations of 2 and 4 μM for 24 h. After treatment, the cells were collected by enzymatic digestion using a trypsin–EDTA solution (Sigma-Aldrich) and transferred to 15 mL tubes. The cell pellet was obtained by centrifugation (5 min at 300*g*). The samples were fixed with ethanol (75% in PBSA) at 4 °C for 24 h. After another centrifugation cycle, the cells were exposed to staining solution for 1 h (PBSA, RNase 1.5 mg/mL, and propidium iodide 90 μg/mL). The analysis was performed using a flow cytometer (Guava easyCyte 8HT, Hayward, CA, USA) with GuavaSoft 2.7 software (1000 events were acquired).

### 2.7 Spheroid Formation Assay

The A549-3D culture model was performed following Friedrich et al.^17^ with modifications. Cells were seeded in 96-well plates previously coated with 2.5% agarose at a density of 2 × 10^3^ cells/well. The plates were maintained in an incubator at 37 °C with a controlled atmosphere (95% air and 5% CO_2_) for 4 days until one spheroid formed in each well. Afterward, the spheroids were treated with CINNAM at 5, 50, and 100 μM, and Cisplatin at 200 μM for 72 h. The 3D cultures were maintained for 9 days after treatment, and spheroids were photographed every 3 days using an EVOS Cell Imaging System (Thermo Fisher Scientific, Waltham, MA, USA) with 4× magnification. The medium was replaced every 48 h. The diameter of each spheroid group (*n* = 12) was analyzed using ImageJ software.

### 2.8 Gene Expression at mRNA Level

Cells were seeded in 35 mm diameter plates at a density of 2 × 10^5^ cells/plate. After collection by enzymatic digestion and centrifugation (300*g* for 5 min at 4 °C), the cell pellet was homogenized in 350 μL of lysis buffer from the RNeasy Mini Kit (Qiagen, Mississauga, ON, Canada). Samples were stored at −80 °C until RNA extraction. Total RNA from each experimental group (*n* = 4) was extracted using the RNeasy Mini Kit (Qiagen, Germantown, MD, USA) according to the manufacturer’s instructions and then eluted in 30 μL of RNase-free water. The total RNA concentration of the samples was measured using the NanoDrop ND 1000 spectrophotometer (NanoDrop Technologies, Wilmington, DE, USA). Then, 1 μg of total RNA was incubated with DNase (1U; Thermo Fisher Scientific) to eliminate possible genomic DNA contamination, followed by reverse transcription (RT) using random primers and the High-Capacity cDNA Reverse Transcription Kit (Thermo Fisher Scientific) according to the manufacturer’s instructions. The reagents were incubated at 25 °C for 10 min, 37 °C for 120 min, and then 85 °C for 5 min for enzyme inactivation.

The mRNA abundance of the target genes (Table 1) was investigated through quantitative polymerase chain reaction (RT-qPCR) using the ABI Prism 7500 thermal cycler (Applied Biosystems, Foster City, CA, USA) and the amplification protocol of the Power Syber Green Master Mix Kit (Thermo Fisher Scientific). Expression values of target genes were normalized using the reference gene *ACTB*. The relative abundance of mRNA for each gene was calculated using the ΔΔ*C*_t_ method with efficiency correction, using a control sample as a calibrator.^18^ The average efficiency values for each gene were calculated from the amplification profile of each sample using the LinRegPCR program.^19^

**Table 1:** Primer Sequences Used in Real-Time PCR Experiments^a^

gene	sequence	reference
*CDKN1A* (p21)	F 5′-CCATAGCCTCTACTGCCACCATC-3′	NM_001291549.1
	R 5′-GTCCAGCGACCTTCCTCATCCA-3′	
*CCND1* (cyclin D1)	F 5′-GGGTTGTGCTACAGATGATAGAG-3′	NM_053056.2
	R 5′-AGACGCCTCCTTTGTGTTAAT-3′	
*CCNE2* (cyclin E2)	F 5′-GGCTATGCTGGAGGAAGTAAAT-3′	NM_057749.2
	R 5′-GCTCTTCGGTGGTGTCATAAT-3′	
*BAX*	F 5′-TTCCTTACGTGTCTGATCAATCC-3′	NM_004324.3
	R 5′-GGGCAGAAGGCACTAATCAA-3′	
*BCL2*	F 5′-CAGAAGTCTGGGAATCGATCTG-3′	NM_000657.2
	R 5′-AATCTTCAGCACTCTCCAGTTATAG-3′	
*ACTB*	F 5′-AGAGCTACGAGCTGCCTGAC-3′	NM_001101.3
	R 5′-AGCACTGTGTTGGCGTACAG-3′	

a *F* = forward primer; R = reverse primer.

### 2.9 Apoptosis Detection

#### 2.9.1 Annexin V/7-AAD Assay

Apoptosis was assessed using the Guava Nexin Reagent Kit (catalog no. 4500-0450, Merk Millipore, Burlington, MA, USA) according to the manufacturer’s instructions. Cells were seeded in 35 mm plates at 2 × 10^5^ cells/well. After adhesion, the cells were treated with CINNAM at 2 and 4 μM for another 24 h. Then, the cells were collected by enzymatic digestion using a trypsin–EDTA solution (Sigma-Aldrich), centrifuged at 300*g* for 5 min, washed in PBSA, and homogenized in a solution containing Annexin V conjugated to PE and 7-AAD. The samples were incubated for 20 min, protected from light at room temperature, and analyzed using a flow cytometer (Guava easyCyte 8HT) with GuavaSoft 2.7 software (5000 events were acquired).

#### 2.9.2 Mitochondrial Membrane Potential (ΔΨm) Analysis by JC-1 Fluorescence

Cellular mitochondrial dysfunction, observed by the loss of mitochondrial membrane potential, was indirectly measured using the fluorescence probe JC-1. For this purpose, the Guava MitoPotential Kit (catalog no. 4500-0250, Merck Millipore, Burlington, MA, USA) was used according to the manufacturer’s instructions. Cells were seeded in 35 mm plates at 2 × 10^5^ cells/well. After adhesion, the cells were treated with CINNAM at 2 and 4 μM for another 24 h. Cells were trypsinized and washed twice with PBS. Subsequently, the cells were labeled with the fluorescent dye JC-1/7-AAD for 30 min at 37 °C. The analysis was performed by flow cytometry (Guava easyCyte 8HT) using GuavaSoft 2.7 software (5000 events were acquired).

#### 2.9.3 Cleaved Caspase-3 Detection

Cells were seeded on coverslips and fixed with 3.7% formaldehyde for 30 min. The cells were permeabilized with 0.5% Triton X-100 for 10 min. After blocking with 1% BSA, cleaved caspase-3 antibody (1:100, Sigma-Aldrich) was incubated overnight at 4 °C. The next day, secondary antirabbit IgG-FITC antibody (catalog no. F9887, Sigma-Aldrich, 1:100) was added to the samples and incubated for 2 h. Nuclei were stained with DAPI, and coverslips were mounted on microscope slides using VectaShield (VectorLabs). All washes were performed with PBS. Analyses were conducted using a confocal microscope (Nikon, Tokyo, Japan, C2).

### 2.10 ROS Detection

Reactive oxygen species (ROS) levels were detected after A549 cells were stained with 2,7-dichlorodihydrofluorescein diacetate (DCFH-DA)^20^ for microscopic observation. Cells were seeded in 35 mm plates on coverslips with an initial inoculum of 2 × 10^5^ cells/plate. After adhesion, the cultures were treated with 4 μM CINNAM for 4 h. Following, cells were incubated for 30 min in complete medium containing MitoTracker (catalog no. M22425, Thermo Fisher Scientific, Waltham, MA, EUA, 500 nM) and DCFH-DA (catalog no. D6883, Sigma-Aldrich, 10 μM) and washed twice with PBS. The slides were immediately analyzed under a fluorescence microscope (Zeiss Axio Scope A1). For flow cytometry quantification, the ROS levels were detected by CellRox Green Fluorescence Dye according to the manufacturer’s instructions. Cells were seeded in 35 mm plates with an initial inoculum of 2 × 10^5^ cells/plate. After adhesion, the cultures were treated with CINNAM (2 and 4 μM) for 4 h. Cells were harvested and incubated in complete medium containing CellRox (catalog n. C10444, Thermo Fisher Scientific, Waltham, MA, EUA, 2.5 μM) at 37 °C for 30 min. The analysis was performed by flow cytometry (Guava easyCyte 8HT) using GuavaSoft 2.7 software. The data are presented as mean ± SD from three independent experiments.

### 2.11 DNA Interaction

The interactions of CINAM with ct-DNA (calf thymus DNA) were analyzed by absorption spectrophotometric analysis using a UV–vis spectrophotometer at room temperature. A standard solution of ct-DNA from Sigma-Aldrich was prepared in tris-HCl buffer (5 mM tris-HCl and 50 mM NaCl, pH 7.4). The concentration of the ct-DNA solution was determined from its absorption intensity at 260 nm using a molar absorption coefficient value of 6600 M^–1^·cm^–1^. The absorption titrations were recorded while keeping the concentration of the complex constant at 1.0 mM in DMSO and increasing the amount of ct-DNA after each addition. The intrinsic equilibrium binding constant (*K*_b_) of the complexes to ct-DNA was obtained by monitoring the changes in the absorption intensity with increasing concentration of ct-DNA and analyzed by regression analysis.

### 2.12 Statistical Analysis

The data presented refer to the mean ± standard deviation (SD) or mean ± standard error of the mean (SEM) of at least three independent experiments, each carried out in triplicate, unless stated otherwise. The results were subjected to one-way analysis of variance (ANOVA) followed by Dunnett’s post-test using GraphPad Prism software 8.0 (GraphPad Software, Inc., San Diego, CA, USA).

## 3 Results

### 3.1 CINNAM is Cytotoxic to A549 and H1299 Cells

The cytotoxicity of ruthenium­(II) complex containing 3,4-methylenedioxy cinnamic acid (CINNAM) was evaluated against lung cancer cells (A549, and H1299) and normal lung fibroblasts (IMR-90). A substantial reduction in cell viability was observed (Figure 3A). CINNAM was cytotoxic to both cancer cell lines, though the degree of responsiveness varied. Notably, the A549 cell line (IC_50_ = 4.31 ± 0.16 μM) was more responsive compared with the H1299 cell line (IC_50_ = 13.50 ± 1.97 μM). Consequently, the A549 cell line was subjected to treatment with free 3,4-methelynedioxy cinnamic acid or the precursor [RuCl_2_(dppb)­(bipy)]. However, the precursor compound was not cytotoxic to any of the cells tested (Figure 3B). Additionally, treatment of A549 cells with cisplatin resulted in an IC_50_ value 14 times higher than that observed for CINNAM under the same experimental conditions (Figure 3B). Normal lung fibroblasts (IMR-90) were less affected by CINNAM (IC_50_ of 23.83 ± 1.08 μM) than tumor cells (Figure 3A,B). Furthermore, we selected concentrations of 2 and 4 μM at CINNAM (IC_50_/2 and IC_50_ values), based on the initial cytotoxicity results to perform subsequent assays.

**Figure 3:**
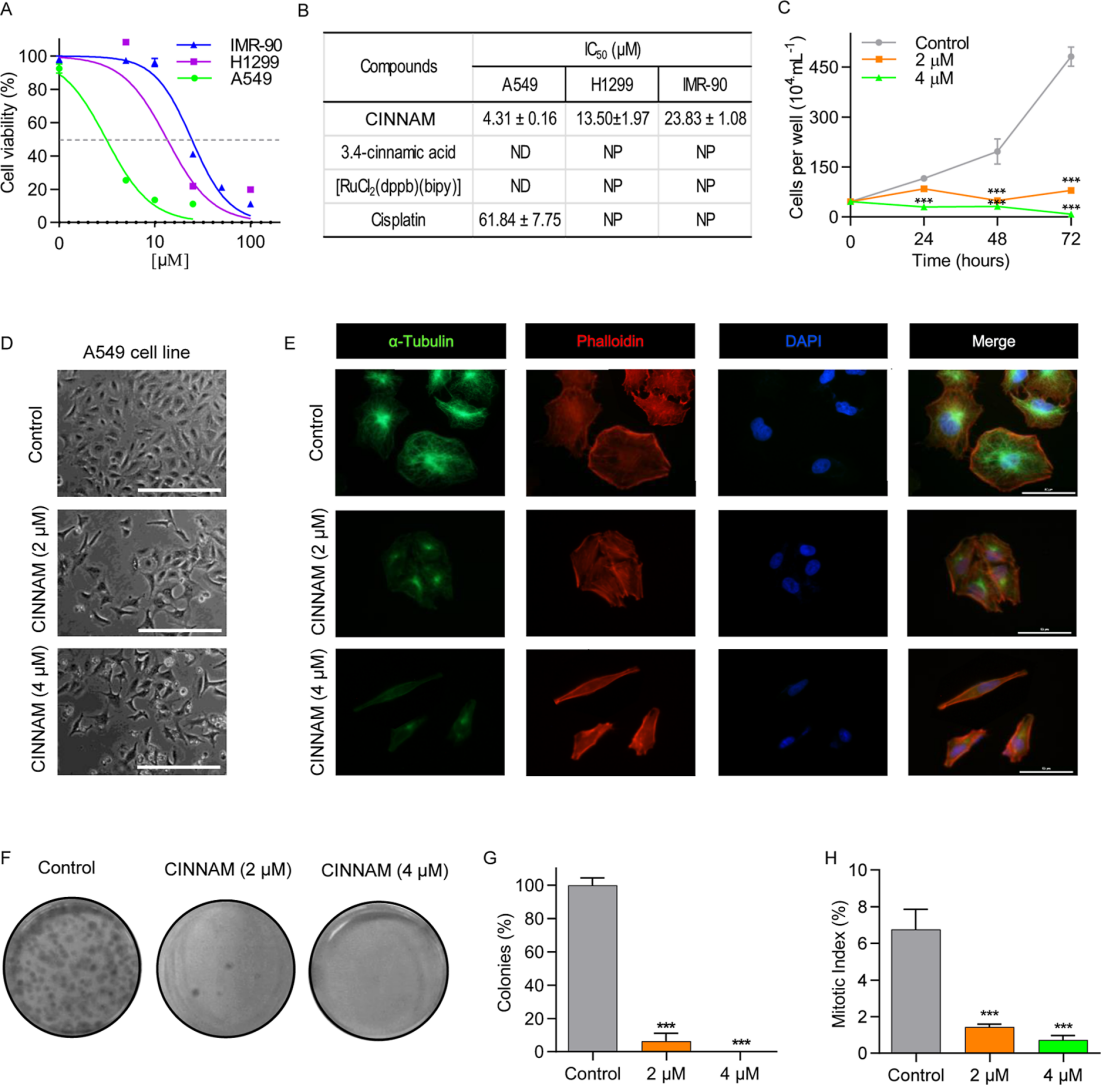
CINNAM is cytotoxic to A549 and H1299 Cells. (A) Dose–response curves determined by MTS. The cells were treated with CINNAM at different concentrations for 24 h. (B) IC_50_ values (μM) determined at 24 h. Cell cultures were treated with CINNAM or its precursor [RuCl_2_(dppb)­(bipy)]. Cisplatin was used as a positive control. ND: not determined, once cell viability was not sufficiently reduced to calculate IC_50_ values. NP: not performed. (C) Cell growth kinetics from 0 to 72 h, determined by Trypan blue exclusion assay. (D) Representative images showing the morphological features of A549 cells treated with 2 or 4 μM of CINNAM for 24 h. Scale bar indicates 200 μm. (E) Fluorescent images showing microtubules (green) and microfilaments (red) distribution pattern of A549 cells in control and CINNAM-treated samples. Nuclei were stained with DAPI. Scale bar indicates 50 μm. (F) Representative images from clonogenic assay. A549 cells were treated with 2 or 4 μM of CINNAM for 24 h and then recovered in fresh medium for 12 days. (G) Quantitative analysis of clonogenic assay. (H) Mitotic index determined after 24 h of treatment by counting mitotic cells in cytological preparations labeled with DAPI and anti-α-tubulin. ***p < 0.001, compared to the control group, according to one-way analysis of variance (ANOVA) followed by Dunnett’s post-test.

The results from the growth kinetics evidenced that in addition to being cytotoxic, CINNAM also inhibited the growth of A549 cells (Figure 3C). Morphological alterations were observed in treated cells, including the presence of cytoplasmic vacuoles and an irregular shape. Cells in suspension and cellular debris were also observed, particularly in cells treated with CINNAM at 4 μM, indicative of high cytotoxicity (Figure 3D). In addition, CINNAM affected the cytoskeleton organization pattern of A549 cells, as demonstrated by drastic disruption of the microtubule and actin filament networks (Figure 3E). Furthermore, CINNAM inhibited the clonogenic capacity of A549 cells. There was a 95% reduction in colony formation in samples treated with 2 μM of CINNAM, while in samples treated with 4 μM of CINNAM, no colony formation was observed at all (Figure 3F,G). CINNAM also decreased the mitotic rate of A549 cells, with mitotic events being 10 times lower in treated cells compared to control cells (Figure 3H).

### 3.2 CINNAM Induces G0/G1 Arrest and Increases p21 (*CDKN1A*) Expression

We selected A549 cells to characterize the molecular mechanisms associated with antiproliferative and cytotoxic potential of CINNAM. Thus, we evaluated how the cell cycle kinetics may have been influenced and observed an increase in the G0/G1 cell population and a reduction in the frequency of cells in the S-phase and G2/M-phase in cultures treated with CINNAM at 2 μM for 24 h (Figure 4A,B). Cells treated with 4 μM of CINNAM also showed a decrease in the S-phase and G2/M-phase. Additionally, CINNAM led to an increased frequency of cells in the sub-G1 population in cultures treated with either 2 or 4 μM. These findings align with the reduced occurrence of BrdU-labeled cells, a specific marker for the S-phase, in CINNAM-treated cultures (Figure 4C,D).

**Figure 4:**
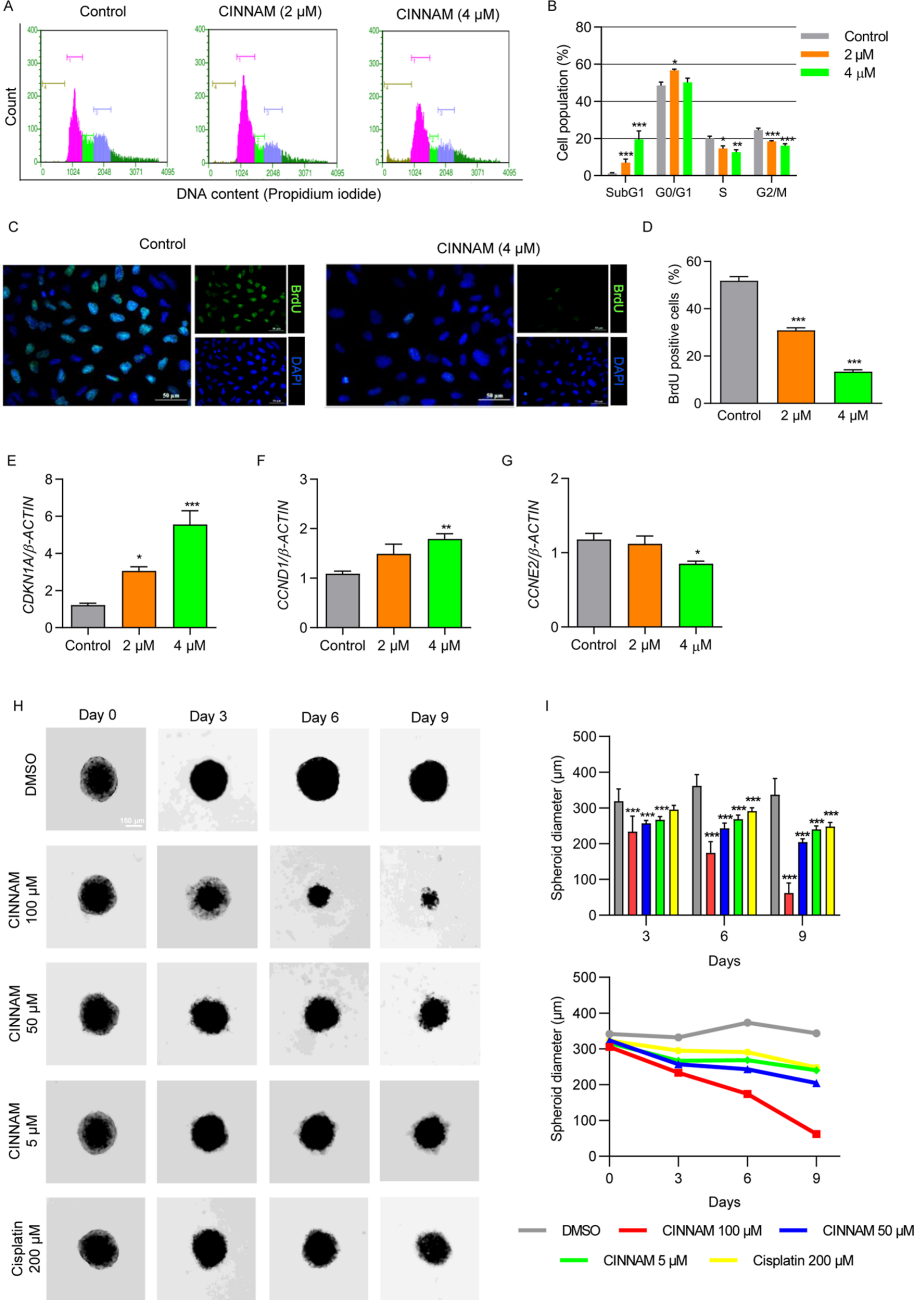
CINNAM induces G0/G1 arrest and increases the expression of *CDKN1A*. (A) Representative histograms obtained from cell cycle analysis using flow cytometry. A549 cells were treated for 24 h with CINNAM at 2 or 4 μM. Sub-G1 (brown), G0/G1 (pink), S (green), G2/M (blue). (B) Quantitative analysis of cell cycle assay. (C,D) Frequencies of cells in S-phase determined by the BrdU incorporation assay, measured after 24 h of treatment. Relative mRNA abundance of (E) *CDKN1A*, (F) *CCND1*, and (G) *CCNE2*. (H) Representative images of A549-spheroids treated with CINNAM at 5, 50, and 100 μM. Cisplatin at 200 μM was used with positive control. Scale bar indicates 150 μm. (I) Quantitative analysis of A549-spheroids (*n* = 12). *p < 0.05, **p < 0.01, ***p < 0.001, compared to the control group, according to one-way analysis of variance (ANOVA) followed by Dunnett’s post-test.

Given that CINNAM induced cell cycle arrest in the G0/G1-phase, we assessed the gene expression of key regulators of G1 progression and the G1/S transition, including *CDKN1A* (p21), *CCND1* (cyclin D1), and *CCNE2* (cyclin E2) using RT-qPCR. CINNAM increased the expression of *CDKN1A* in A549 cells, with mRNA abundance 2.5 and 4.6 times higher in cultures treated with 2 and 4 μM for 24 h, respectively (Figure 4E). Additionally, CINNAM at 4 μM increased the mRNA abundance of *CCND1* and reduced the mRNA abundance of *CCNE2* (Figure 4F,G). These findings suggest that the modulation of *CDKN1A* and G1/S-phase cyclins may be associated with the antiproliferative activity of CINNAM in A549 cells.

### 3.3 CINNAM Reduces the Proliferation of A549-Spheroids

Since three-dimensional culture has emerged as an essential in vitro model for cancer research, particularly for recapitulating the architecture of tumors in vivo,^17^ we performed experiments using A549 spheroids to evaluate the effects of CINNAM in this model. CINNAM significantly reduced the diameter of A549 spheroids after 9 days of treatment (Figure 4H,I), indicating a sustained inhibition of cell proliferation. This reduction in spheroid size is likely a result of CINNAM’s long-term effects on A549 cell growth.

### 3.4 CINNAM Induces Apoptosis via ROS Generation in A549 Cells

Cell cycle analysis revealed a significant increase in the sub-G1 population, indicating the induction of cell death. To assess the pro-apoptotic potential of CINNAM, annexin V flow cytometry and expression analysis of the pro-apoptotic gene *BAX* and antiapoptotic gene *BCL2* were conducted using RT-qPCR. Treatment with 2 or 4 μM of CINNAM for 24 h increased the frequency of annexin V-positive cells (Figure 5A,B). Additionally, CINNAM increased the mRNA abundance of the pro-apoptotic gene *BAX* in A549 cells after 24 h (Figure 5C). Immunofluorescence analysis further showed that CINNAM increased the frequency of cells positive for cleaved caspase-3 (Figure 5D,E). These results suggest that the observed cell death is likely due to the induction of apoptosis.

**Figure 5:**
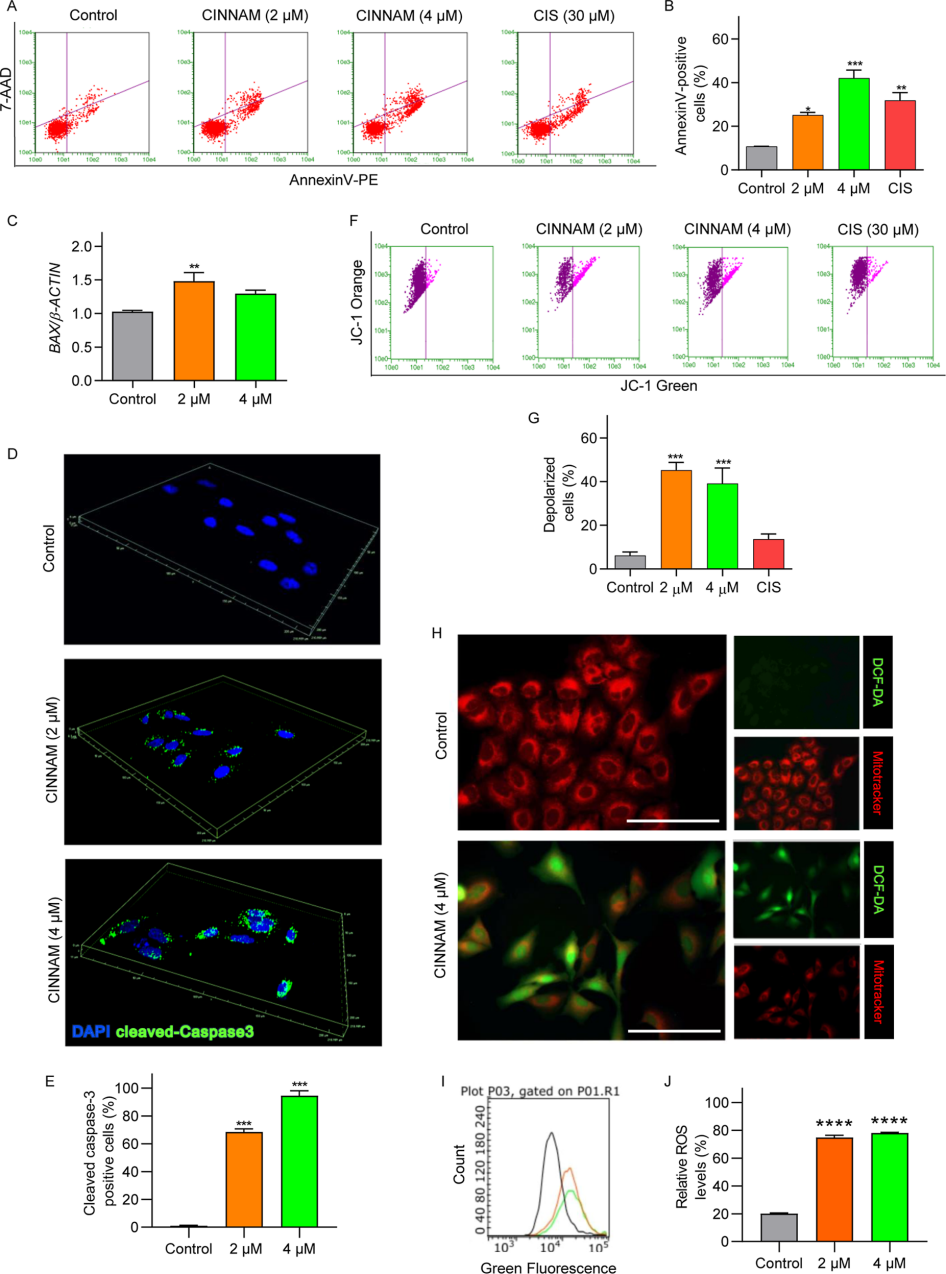
CINNAM induces apoptosis via ROS generation in A549 cells. (A) Representative dot plots obtained from the analysis of Annexin V-PE/7-AAD using flow cytometry. The lower left quadrants show viable cells, which are negative for both 7-AAD and Annexin V-PE. The upper left quadrants contain nonviable, necrotic cells, negative for Annexin V-PE and positive for 7-AAD. The lower right quadrants represent cells in early apoptosis, which are Annexin V-PE positive and 7-AAD negative. The upper right quadrants represent cells in late apoptosis, positive for both Annexin V-PE and 7-AAD. (B) Quantitative analysis of annexin V assay. (C) Relative gene expression of *BAX*. (D) Representative image exhibiting the staining pattern for cleaved caspase-3 immunodection (green). (E) Quantitative analysis of cells positive for cleaved-caspase 3. (F) Representative dot plots and analysis of mitochondrial membrane potential using JC-1 as a fluorescent probe. (G) Quantitative analysis of mitochondrial membrane potential assay. (H) Detection of ROS (green) with 2,7-dichlorodihydrofluorescein diacetate (DCFH-DA); MitoTracker Red Fluorescence dye was used as a marker of health mitochondria marker. Scale bar indicates 100 μm. (I) Measurement of ROS levels by CellRox Green using flow cytometry. A549 cell were treated with CINNAM for 4 h. The solid lines in the representative histogram correspond to control (0.1% DMSO, v/v) (gray), CINNAM at 2 μM (orange) and, CINNAM at 4 μM (green) groups, respectively. (J) Quantitative analysis of CellRox green assay. *p < 0.05, **p < 0.01, ***p < 0.001, compared to the control group, according to one-way analysis of variance (ANOVA) followed by Dunnett’s post-test.

CINNAM was able to trigger the activation of the intrinsic apoptotic pathway in A549 cells. To support these findings, we used the fluorescent probe JC-1 to measure changes in mitochondrial membrane potential (MMP). The analysis revealed an increase in the percentage of cells with lower Δψm (a higher ratio of green to red fluorescence) in cells treated with CINNAM (Figure 5F,G). To determine whether these changes in MMP were induced by ROS, we measured the colocalization of the ROS marker 2,7-dichlorodihydrofluorescein diacetate (DCFH-DA) with the mitochondrial marker MitoTracker Red fluorescence dye. Cells treated with 4 μM of CINNAM exhibited a marked increase in green fluorescence compared to control cultures, indicating elevated ROS levels (Figure 5H). Furthermore, CINNAM altered the mitochondrial organization pattern in treated cells (Figure 5H). In addition, by CellRox Green Flow Cytometry, an expressive increased of intracellular ROS levels was observed in cells treated with CINNAM (2, and 4 μM) (Figure 5I,J). Collectively, these results suggest that the antiproliferative and pro-apoptotic activity of CINNAM may be associated with mitochondrial alterations and ROS generation in A549 cells.

### 3.5 DNA-Binding: UV–Vis Spectrophotometric Titration

The interaction with DNA may indicate a potential target for the complex.^21,22^ So, the UV–visible absorption spectroscopy is a useful direct method for determining the DNA binding constants of metal complexes that can interact at distinct binding sites (groove binding outside of the DNA helix along the major or minor groove, electrostatic binding to a phosphate group, and intercalation). Shifts in the spectrum provide evidence of such complex interaction with DNA.^23^

Upon adding the solution of ct-DNA in the complex, a decrease in the absorption intensity (hypochromism) was observed (Figure 6). This hypochromism, combined with the absence of a bathochromic shift, indicates that the complex interacts with DNA via groove binding.^24^

**Figure 6:**
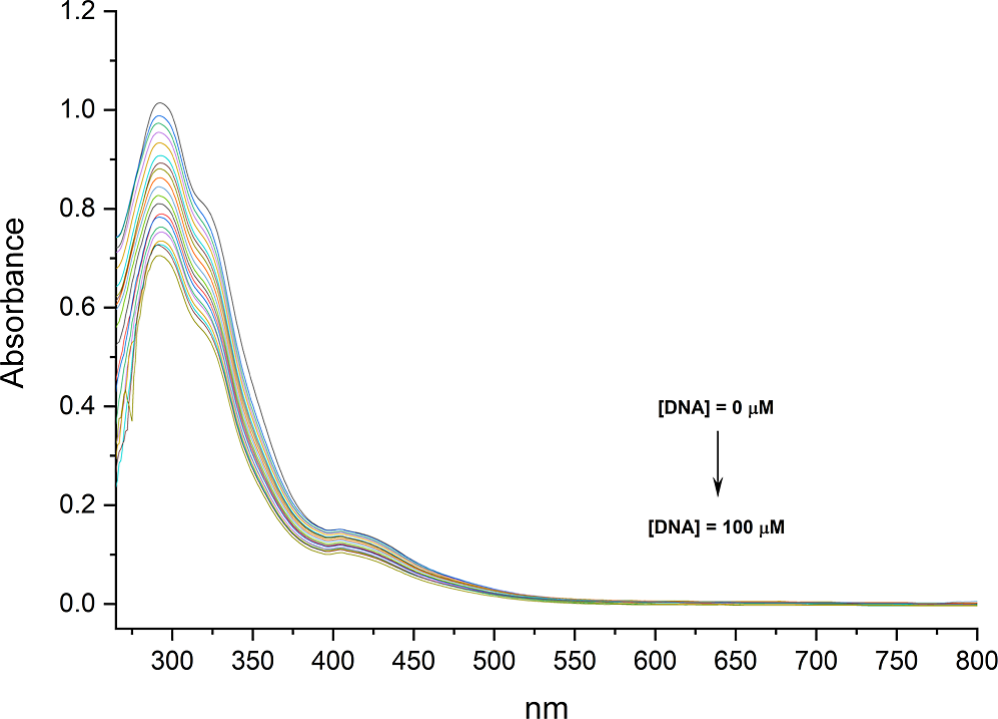
Changes in the electronic absorption spectra of the complex with increasing concentrations of ct-DNA.

The strength of this interaction was quantified by the DNA binding constant (*K*_b_), calculated using the Benesi–Hildebrand method.^25^ The compound exhibited a *K*_b_ value of 4.3 ± 2.4 × 10^5^ M^–1^ and a hypochromic percentage (% H) of 11.6%. Based on the data, the binding constants (*K*_b_) are in a smaller order of magnitude when compared to ruthenium complexes that interact with DNA via grooves.^26^ Therefore, based on the *K*_b_ values, the complexes present intermediate affinity for DNA.

## 4 Discussion

The results of our study underscore the significant potential of ruthenium­(II) complex containing 3,4-methylenedioxy cinnamic acid (CINNAM) as a potent agent against NSCLC-derived cancer cells. CINNAM was highly cytotoxic, particularly to A549 cells, which exhibit a gain-of-function in *KRAS* and a loss-of-function in *KEAP1*.^27,28^ The H1299 cells harbor a gain-of-function in *NRAS* and a large deletion in *TP53*.^29^ Our findings indicate that the CINNAM compound may influence the proliferative behavior of tumor cells by affecting the RAS pathway and/or redox metabolism.^30^ However, apparently, the higher responsiveness of A549 to CINNAM compared to H1299 seems to be related to the NRF2 signaling pathway. The A549 cell line exhibits aberrantly active NRF2 due to a somatic mutation of the KEAP1 gene at G333C and epigenetic alteration by methylation in the KEAP1 promoter.^31^ Further studies will be performed to validate this hypothesis. Our findings show that CINAM has anticancer potential, which should be further investigated to characterize its molecular targets.

The precursor [RuCl_2_(dppb)­(bipy)] did not exhibit cytotoxicity against NSCLC, highlighting the critical role of 3,4-methyledioxy cinnamic acid coordination in the ruthenium complex for its pharmacological activity. Additionally, CINNAM demonstrated an IC_50_ that was 14 times lower than that of cisplatin, indicating significantly greater potency under similar experimental conditions. CINNAM also demonstrated notable selectivity for tumor cells, with an IC_50_ for normal lung fibroblasts (IMR-90) being 5.52 times higher than for A549 cells, suggesting lower toxicity to healthy tissues. These promising results show that CINNAM is a selective anticancer agent, warranting further detailed characterization of its mechanism of action.

We demonstrated that CINNAM not only exerted significant cytotoxic effects on A549 cells, but also effectively inhibited their proliferation in both the short and long-term. The inhibition of clonogenic capacity, evidenced by a dramatic reduction in colony formation and mitosis in the 2D model, aligns with the reduction in A549 spheroid proliferation, highlights CINNAM’s ability to disrupt long-term survival. These findings align with other studies that identify the cytotoxic and antiproliferative potential of ruthenium complexes against cancer cells. For instance, Bomfim et al.^32^ demonstrated that Ruthenium­(II) complexes with 6-methyl-2-thiouracil reduced cell proliferation and increased phosphatidylserine externalization, activation of caspase-3, -8, and -9, and loss of mitochondrial transmembrane potential in HL-60 cells. Cervinka et al.^33^ tested ruthenium­(II)­tris-pyrazolylmethane complexes against various cancer cell lines. They observed that the complexes showed micromolar potency against cancer cells, demonstrated significant selectivity over noncancerous cells, and effectively disrupted mitochondrial homeostasis, leading to apoptosis.

The cell cycle analysis demonstrated that CINNAM exerts its antiproliferative effects on A549 lung cancer cells, at least in part, by inducing G0/G1 cell cycle arrest. By significantly increasing the proportion of cells in the G0/G1 phase and reducing the number of cells in the S and G2/M phases, CINNAM effectively halted cell cycle progression, thereby inhibiting cell proliferation. The upregulation of *CDKN1A* (p21), a critical cyclin-dependent kinase inhibitor, further supports this mechanism, as p21 is known to enforce G1 arrest by inhibiting the activity of cyclin-CDK complexes necessary for the G1/S transition.^34,35^ The simultaneous increase in cyclin D1 (*CCND1*) expression and decrease in cyclin E2 (*CCNE2*) expression suggest a complex modulation of cell cycle regulators. CINNAM potentially promotes a state where cells are primed for G1 arrest but unable to progress through the cycle. This blockage of cell cycle progression prevents the replication of cancer cells, contributing to the overall cytotoxic effect of CINAM.

The effect of ruthenium complexes on cell cycle arrest in tumor cells has also been evidenced in lung tumor cells,^36^ prostate cancer cells,^37^ breast cancer cells,^38^ and hepatocarcinoma cells.^39^ In the broader context of cancer treatment, these findings underscore the potential of CINNAM as a therapeutic agent targeting the proliferative capacity of tumor cells by disrupting key regulatory pathways of the cell cycle.

The findings reveal that CINNAM effectively induces apoptosis in A549 lung cancer cells by triggering the intrinsic apoptotic pathway, with mitochondrial dysfunction and ROS generation playing critical roles in this process. Increased sub-G1 population, alongside the elevated presence of annexin V-positive cells, strongly indicates that CINNAM drives cell death through apoptosis. This is further corroborated by the upregulation of the pro-apoptotic gene BAX and the increased detection of cleaved caspase-3, a key executioner of apoptosis.^40^ The involvement of the intrinsic pathway is particularly significant, as it highlights CINNAM’s ability to disrupt mitochondrial homeostasis, leading to a cascade of events that culminate in programmed cell death.

CINNAM’s impact on mitochondrial membrane potential (MMP) is especially noteworthy. The observed decrease in MMP, evidenced by a higher ratio of green to red fluorescence in JC-1-stained cells, suggests a loss of mitochondrial integrity, which is a hallmark of the intrinsic apoptotic pathway.^41,42^ This disruption of MMP is closely associated with the generation of ROS,^43^ as indicated by the marked increase in ROS levels in CINNAM-treated cells. The colocalization of ROS with mitochondrial markers implies that CINNAM induces oxidative stress specifically within the mitochondria, further exacerbating mitochondrial dysfunction and promoting apoptosis.

The link between ROS generation and apoptosis in cancer cells is well-established, with excessive ROS leading to oxidative damage, activation of pro-apoptotic signaling pathways, and eventual cell death.^44,45^ CINNAM’s ability to elevate ROS levels within the mitochondria suggests that it may selectively target cancer cells by exploiting their often already elevated oxidative stress levels,^44^ pushing them beyond their threshold for survival. This selective induction of apoptosis through mitochondrial targeting and ROS generation underscores CINNAM’s potential as a promising therapeutic agent for lung cancer treatment.

Fandzloch et al.^46^ demonstrated that MCF-7 breast cancer cells exhibit high levels of intracellular ROS after treatment with ruthenium­(III) complexes, which is followed by apoptosis. Similar results were found by Chen et al.,^47^ who showed that Ruthenium­(II) salicylate selectively inhibits the activity of thioredoxin reductase (TrxR), a major component of the thioredoxin system, thereby promoting the generation and accumulation of ROS. TrxR inhibition was critical for triggering mitochondrial dysfunction and apoptosis in A549 lung cancer cells.

Moreover, these findings align with broader trends in cancer therapy, where targeting mitochondrial function and exploiting oxidative stress are emerging as effective strategies for inducing cancer cell death while minimizing damage to normal cells.^48^ CINNAM’s dual action in disrupting mitochondrial membrane potential and contributing for ROS generation become it a potent antitumor agent capable of selectively eliminating cancer cells. Further research into the precise molecular mechanisms by which CINNAM modulates mitochondrial function and induces ROS generation could provide deeper insights into its therapeutic potential. Such studies could pave the way for the development of novel cancer treatments based on similar mechanisms.

## 5 Conclusion

In this study, the cytotoxic potential ruthenium­(II) complex containing 3,4-methylenedioxy cinnamic acid (CINNAM) was evaluated against nonsmall cell lung cancer (NSCLC) cell lines with different genetic profiles (A549, and H1299). CINNAM effectively inhibited proliferation in the A549 cell line. The antiproliferative activity of CINNAM was associated with its ability to modulate cell cycle regulators, including *CDKN1A* (p21) and G1/S cyclins (cyclin D, and E). Additionally, its cytotoxic activity was likely due to its capacity to promote apoptosis by mitochondrial disruption. CINNAM exhibited pro-oxidant activity, contributing to its antiproliferative and pro-apoptotic effects on A549 cells. Additionally, DNA-binding experiments yielded constants of 10^5^ M^–1^ (*K*_b_ = 4.3 ± 2.4 × 10^5^ M^–1^) and demonstrated that CINAM has intermediate affinity for DNA. Our findings suggest that CINNAM is a promising candidate for further in vivo studies that reinforce its antitumor potential against lung cancer.

## Supplementary Material



## Notes

Figures S1–S7 present the complete characterization of the CINNAM compound. Figure S1 shows the HPLC chromatogram, confirming the compound’s purity. Figures S2, S3, and S4 provides insights into the elemental analysis through ^31^P­{^1^H} NMR spectrum in CH_2_Cl_2_ + D_2_O capillary, and ^1^H and ^13^C­{^1^H} NMR spectra in CDCl_3_, respectively. The UV–vis spectrum in DMSO (Figure S5) highlights the compound’s electronic transitions, while the infrared spectrum (Figure S6) confirms the presence of key functional groups. Finally, Figure S7 displays the cyclic voltammetry profile, which indicates the electrochemical stability of the coordination complex (PDF)
