# Supplementary material for: Ruthenium(II) Complex with 3,4-Methylenedioxy Cinnamic Acid Induces Cell Cycle Arrest at G0/G1 and Apoptosis via ROS Generation and Bioenergetics Disruption in Non-Small Cell Lung Cancer Cells
Source: ACS Omega. 2025 Jul 1;10(27):28956–68. doi: 10.1021/acsomega.5c00526 (PMC12268433; doi:10.1021/acsomega.5c00526)
Supplement: Supplementary file 1 [file ao5c00526_si_001.pdf]

Ruthenium (II) Complex with 3,4-methylenedioxy cinnamic Acid Induces Cell Cycle Arrest at G0/G1 and Apoptosis via ROS Generation and Bioenergetics Disruption in Non-Small Cell Lung Cancer Cells

Guilherme Álvaro Ferreira-Silva[1], Caio Cesar Candido[2], Graciana Yokota Garavelli[1], Carolina Girotto Pressete[1], Ester Siqueira Caixeta[1], Angelica Ellen Graminha[3], Marília Imaculada Frazão Barbosa[2], Antônio Carlos Doriguetto[2], Marisa Ionta[1], Alexandre Ferro Aissa[1]**

AUTHOR ADDRESSES

[1] Institute of Biomedical Science, Federal University of Alfenas, 37130-000, Alfenas, MG, Brazil

[2] Institute of Chemistry, Federal University of Alfenas, 37130-001, Alfenas, MG, Brazil.

[3] Institute of Chemistry, São Paulo State University, 14800-060, Araraquara, SP, Brazil.

Summary

Figure S1. HPLC analysis of CINNAM

Figure S2.: $^{31}P\{^1H\}$ spectroscopy of CINNAM, in CH_2Cl_2 + D_2O capillary

Figure S3. ^1H spectroscopy of CINNAM, in $CDCl_3$

Figure S4. $^{13}C\{^1H\}$ spectroscopy of CINNAM, in $CDCl_3$

Figure S5. UV-Vis spectroscopy of CINNAM, in DMSO

Figure S6. Infrared spectroscopy of CINNAM

Figure S7. Cyclic voltammetry of CINNAM

1 Sequencia Caio 10 03

Sample Name:	**Sequencia Caio 10 03**	*Injection Volume:*	**10,0**
Vial Number:	**RA1**	*Channel:*	**UV_VIS_3**
Sample Type:	**unknown**	*Wavelength:*	**330.0**
Control Program:	**Programa Caio 10 03**	*Bandwidth:*	**2**
Quantif. Method:	**Metodo Caio 10 03**	*Dilution Factor:*	**1,0000**
Recording Time:	**11/3/2025 15:05**	*Sample Weight:*	**1,0000**
Run Time (min):	**30,00**	*Sample Amount:*	**1,0000**

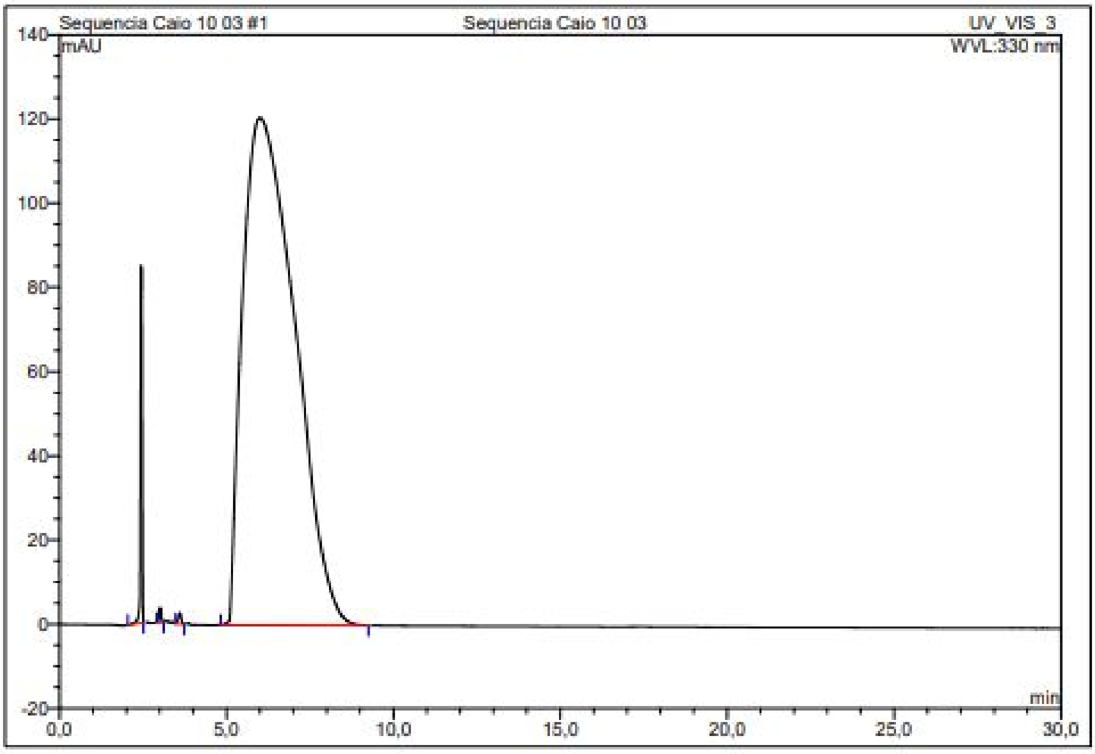

No.	Ret.Time min	Peak Name	Height mAU	Area mAU*min	Rel.Area %	Amount	Type
1	2,45	n.a.	84,735	4,100	1,84	n.a.	BMB
2	3,01	n.a.	3,537	0,271	0,12	n.a.	BMB
3	3,60	n.a.	2,684	0,264	0,12	n.a.	BMB
4	6,01	n.a.	120,484	217,731	97,92	n.a.	BMB
Total:			211,440	222,366	100,00	0,000	

Figure S1: HPLC analysis of CINNAM, gradient: methanol: water (90:10)

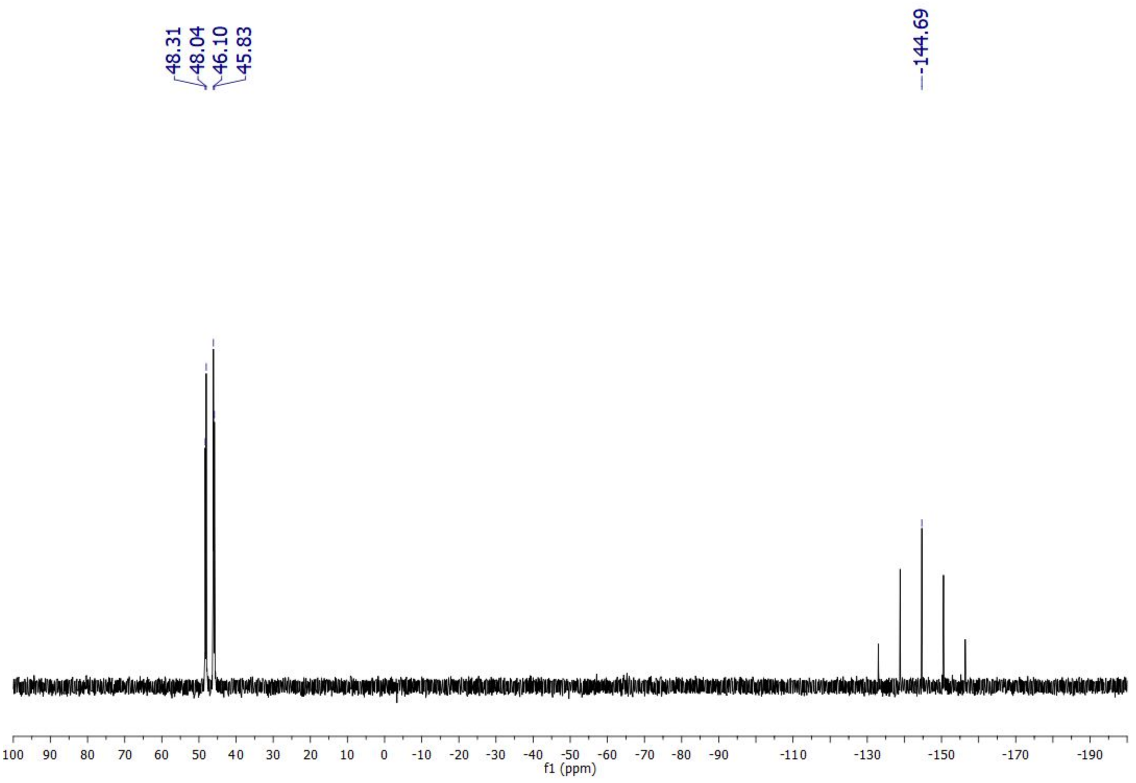

Figure S2: $^{31}P\{^{1}H\}$ spectroscopy of CINNAM, in CH_2Cl_2 + D_2O capillary

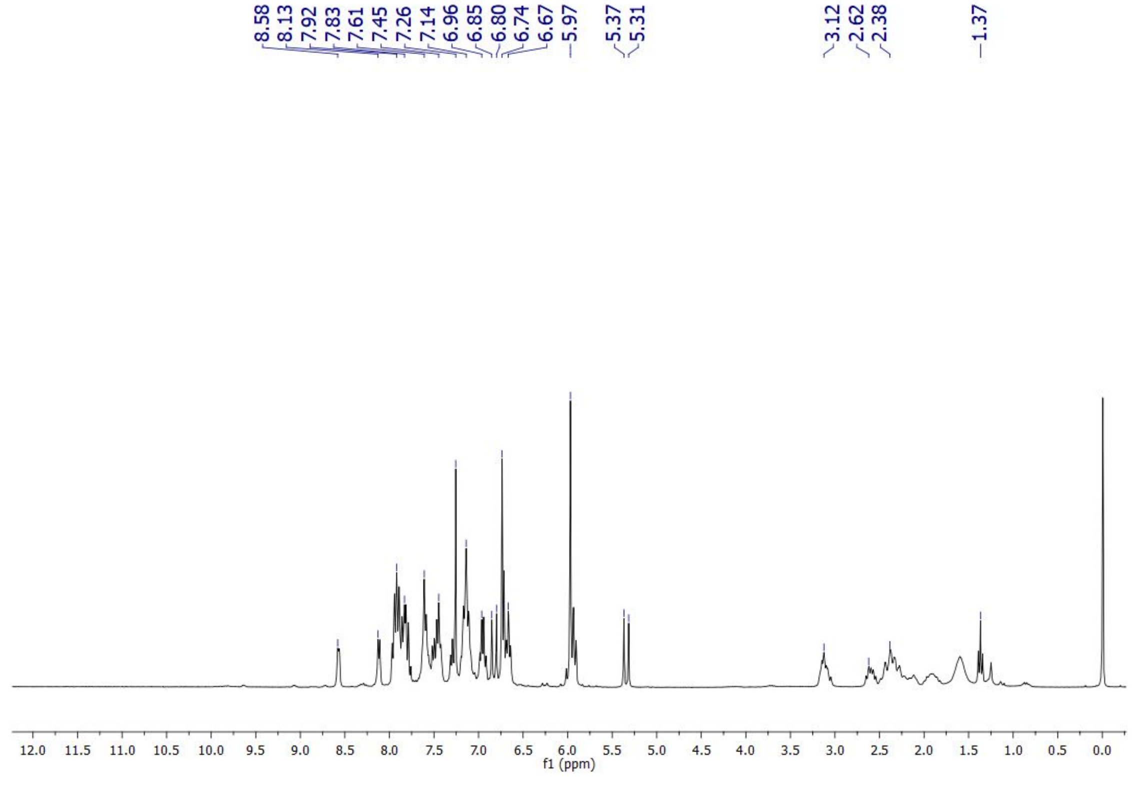

Figure S3: ^{1}H spectroscopy of CINNAM, in $CDCl_3$

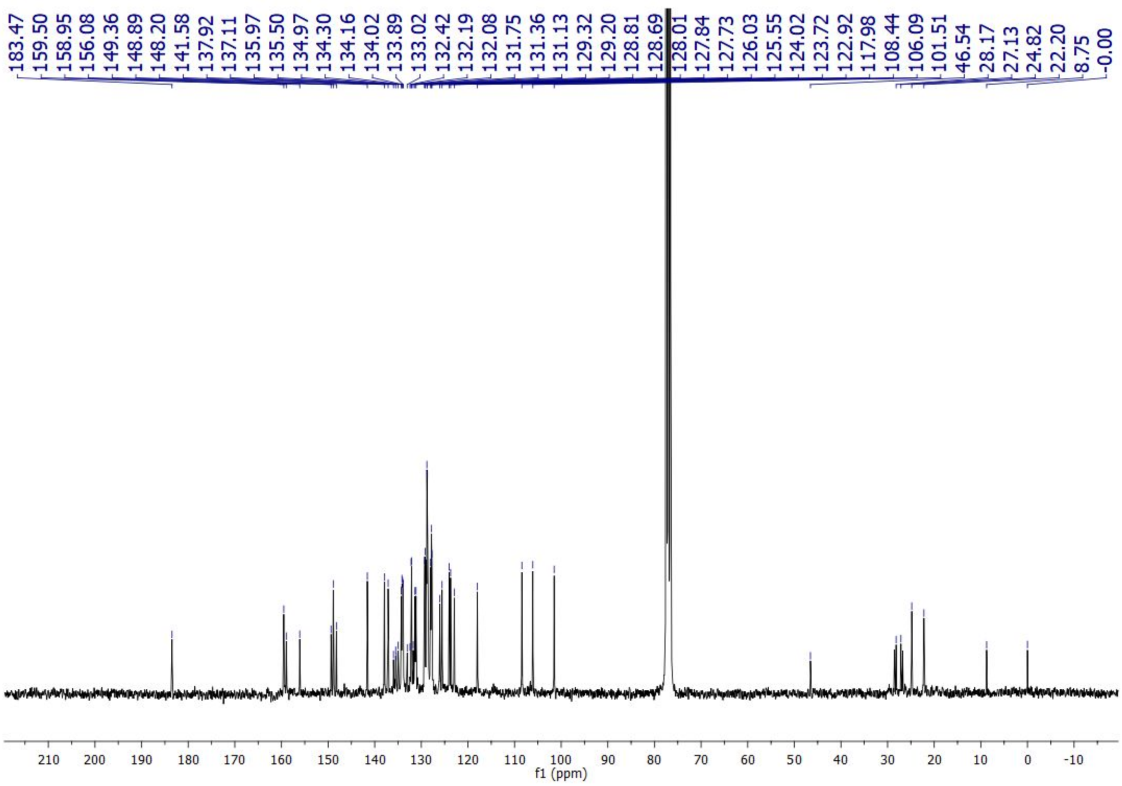

Figure S4: $^{13}C\{^{1}H\}$ spectroscopy of CINNAM, in $CDCl_3$

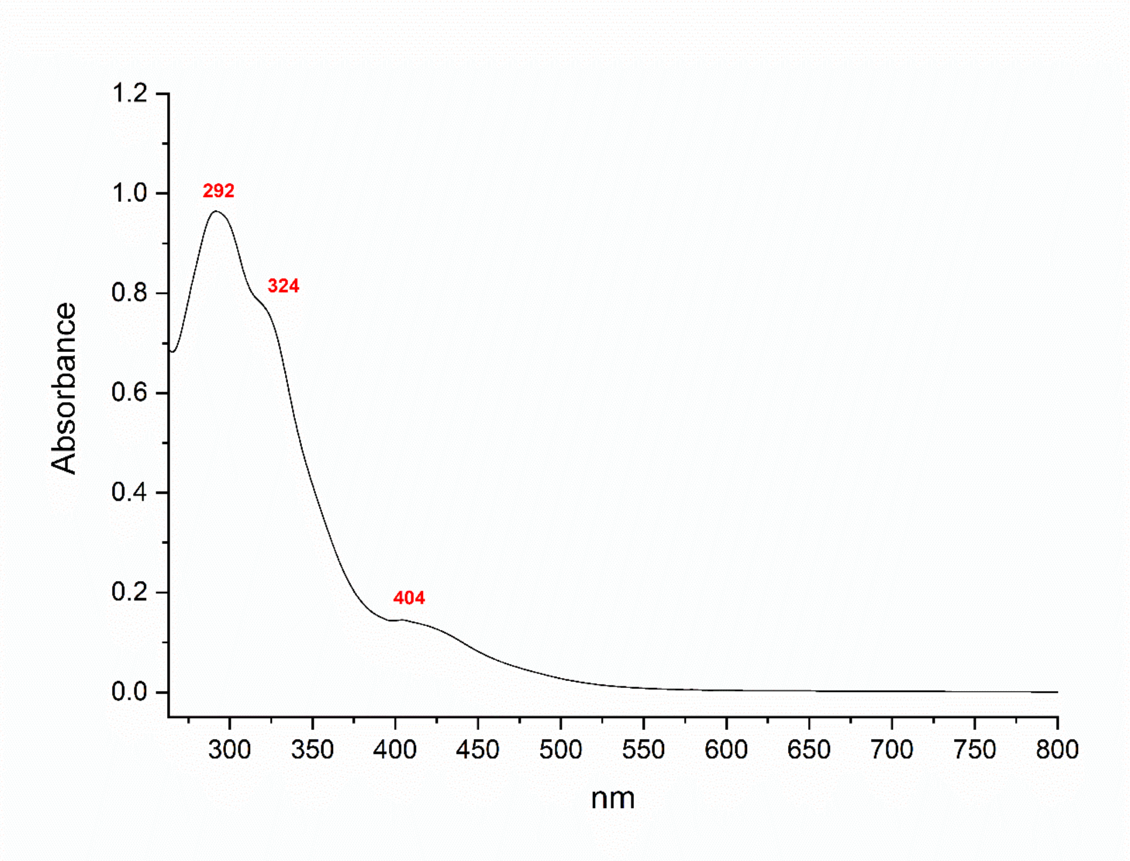

Figure S5: UV-Vis spectroscopy of CINNAM, in DMSO

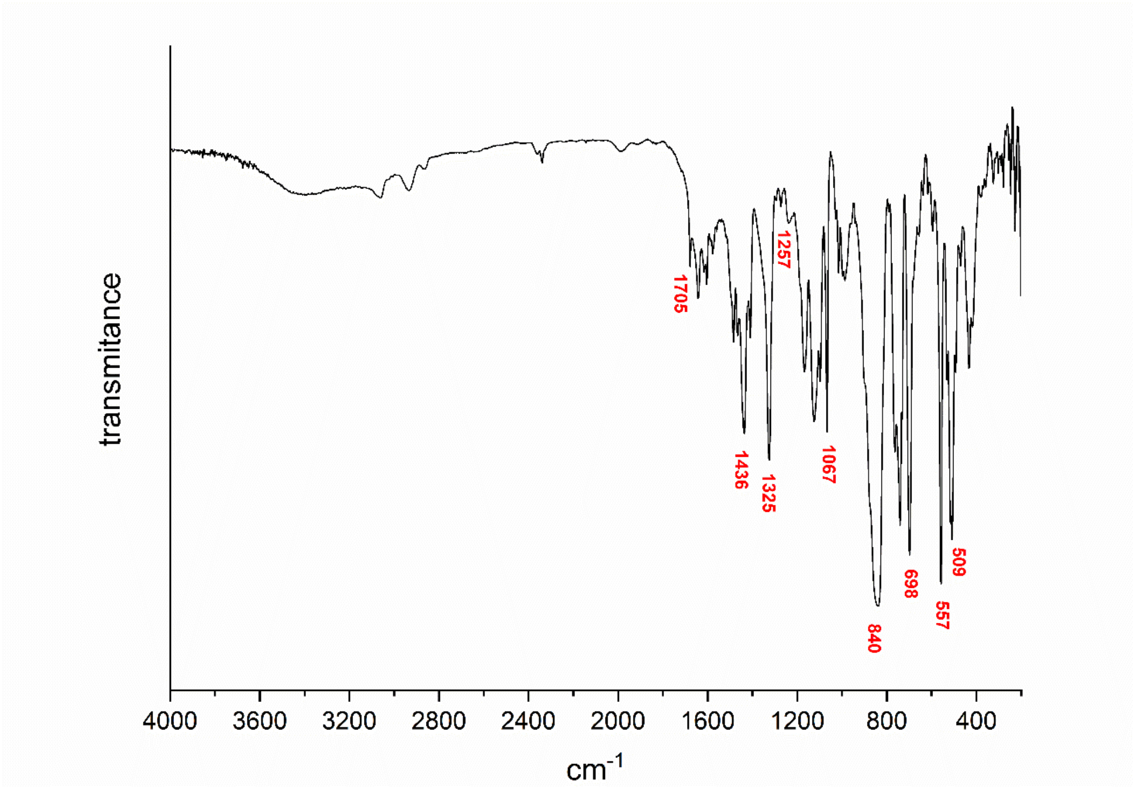

Figure S6: Infrared spectroscopy of CINNAM

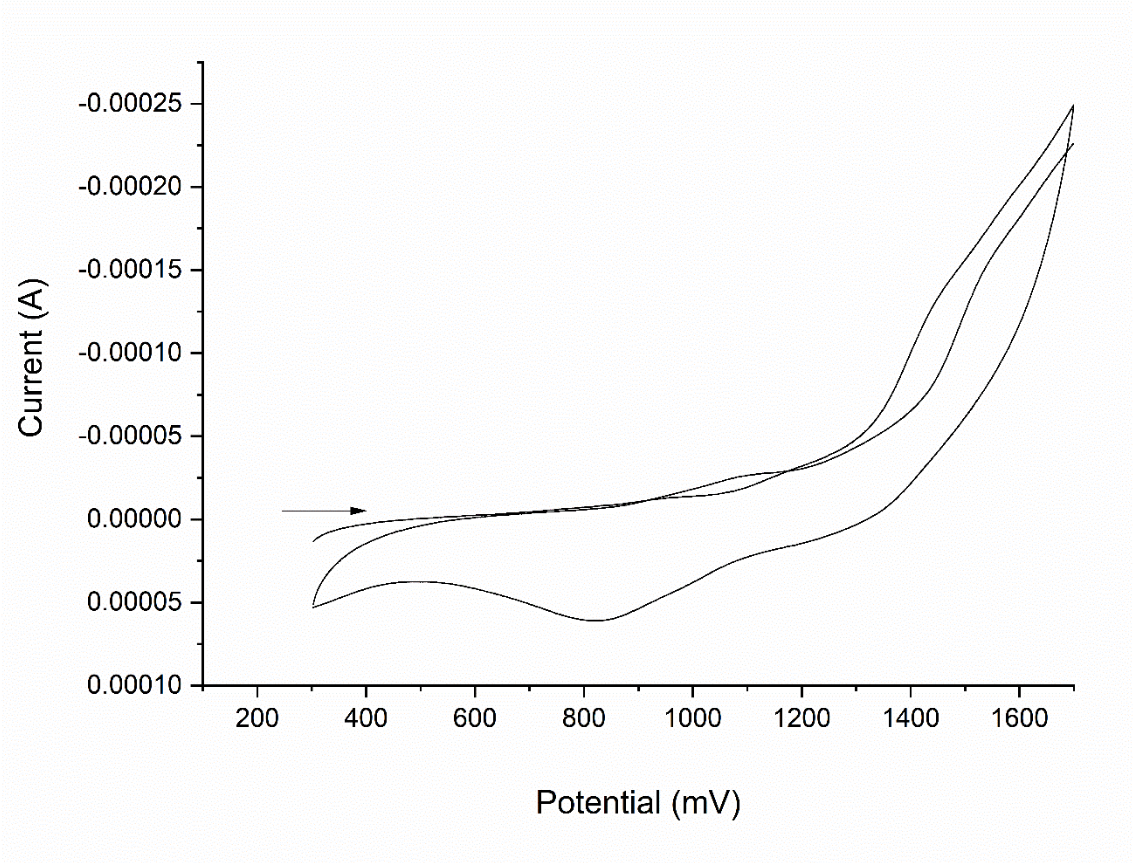

Figure S7: Cyclic voltammetry of CINNAM
